# Prognostic models for alcoholic hepatitis

**DOI:** 10.1186/s40364-015-0046-z

**Published:** 2015-07-21

**Authors:** Erik Rahimi, Jen-Jung Pan

**Affiliations:** Division of Gastroenterology, Hepatology and Nutrition, University of Texas Medical School at Houston, 6431 Fannin Street, MSB 4.234, Houston, TX 77030 USA

**Keywords:** Alcoholic hepatitis, Prognostic score, Mortality, Risk

## Abstract

Alcoholic hepatitis (AH) is caused by acute inflammation of the liver in patients that consume excessive amounts of alcohol, usually in a background of cirrhosis. AH can range from mild to severe, life threatening disease with a high rate of short and long-term mortality. Prognostic models have been used to estimate mortality in order to identify those that may benefit from corticosteroids or pentoxifylline. This review focuses on the different prognostic models proposed. While limitations of the prognostic models exist, combining models may be beneficial in order to identify responders to therapy versus non-responders.

## Introduction

Alcoholic hepatitis (AH) is a syndrome due to acute inflammation of the liver in patients that consume excessive amounts of alcohol [1]. Rapid onset of jaundice due to parenchymal injury ranging from mild to severe, life-threatening disease usually in the background of concomitant cirrhosis is the hallmark presentation of AH [2]. Along with jaundice, varying symptoms and signs consist of fever, encephalopathy, abdominal distress, ascites, varices, anemia, leukocytosis, coagulopathy, and a ratio of serum aspartate aminotransferase (AST) to alanine aminotransferase (ALT) greater than 2, which rarely exceeds 300 IU per liter, are seen [3–5]. Although it is a treatable form of alcoholic liver disease, there is a high rate of short and long-term mortality. The overall inpatient mortality was 6.6 %–6.8 % for acute AH and 13.6 % for chronic AH in population-based studies [1,6]. In a Danish study, the 28-day mortality was 15 %, 84-day mortality was 24 %, and 5-year mortality was 56 % with a further increase of mortality in cirrhotic patients [7]. Another study from the United Kingdom estimated a 5-year survival of 31.8 % after index hospitalization with severe AH. Abstinence from alcohol was the only independent predictor of long-term survival [8].

While mild AH usually enjoys a good prognosis with alcohol abstinence only, severe AH is associated with high mortality rates and requires additional treatment. Corticosteroids and pentoxifylline are the main pharmacological treatment options that have shown to improve short-term survival, although the overall outcomes are still poor and can cause potential adverse events [9]. Estimating prognosis and identifying those who will need treatment is therefore extremely important. Prognostic models are developed by combining two or more items of patient data into a statistical model to potentially predict clinical outcomes [10]. Non-invasive scoring systems are important for prognosis as more invasive testing such as liver biopsies can lead to increased morbidity [11]. In this article, we review several scoring systems that are available to assess severity and prognosis of AH.

### Maddrey discriminant function

The American Association for the Study of Liver Diseases (AASLD) practice guidelines recommend that patients suspected of having AH should have their outcome risk stratified using the discriminant function (DF), along with other available clinical data [2]. In a placebo-controlled study assessing benefits of prednisolone in AH, Maddrey et al. in 1978 first yielded the DF based on prothrombin time (PT) and serum bilirubin concentration that identified patients with a significant risk for early mortality [12]. This was later modified in 1989 to the modified DF (or mDF) = 4.6 (patient’s PT in seconds- control PT in seconds) + total bilirubin (mg/dL) that is used today. Those patients with mDF >32 were considered to have severe AH. Patients with an elevated mDF and/or with encephalopathy that received corticosteroid therapy, showed a 28-day mortality of 6 % in the treatment group compared to 35 % in the placebo group [13]. On retrospective analysis, DF is a valid index of severity of disease identifying those at 50 % risk of death within 2 months [14]. DF has an inadequate specificity of <40 %–62 % and a sensitivity of 67 %–100 % for short-term (30 day) mortality [15,16]. The inaccuracy in using DF may be accounted for the cut-off point of 32, and had been the basis of debate on differing effectiveness of corticosteroid treatment. Higher cut-off values have been proposed such as cut-offs of 33, 37, 41, 42, or 44 to increase the specificity [15–18]. However, DF scores less than 32 have also been shown to be associated with a relatively high 28-day mortality of 16.7 %. DF was shown to have a poor diagnostic performance since it can only predict mortality or survival 66.6 % of the time [15]. A drawback of using DF is the variable results of PT across different laboratories. The PT test relies on thromboplastin, a variable reagent, making inter-laboratory results vary greatly [19]. Due to the aforementioned limitations of DF, several other prognostic models as listed below have therefore been developed.

### Model for end-stage liver disease (MELD) score

The MELD score is calculated using bilirubin, creatinine and international normalized ratio (INR) levels. The objective parameters were originally used to predict early death following elective transjugular intrahepatic portosystemic shunts (TIPS) [20]. The use of objective and reproducible data was subsequently shown to be a reliable measure of short-term mortality risk in patients with end-stage liver disease independent of complications of portal hypertension, and is used as a disease severity index to determine organ allocation priorities [21]. Several studies have used the MELD score to assess disease severity in AH. In a study by Sheth et al [18], the MELD score had a similar performance as the DF in predicting mortality at 30 days. The sensitivity and specificity in predicting 30-day mortality was 86 % and 82 %, respectively, for MELD scores >11 compared to 86 % and 48 %, respectively, when DF was greater than 32. The utility of predicting mortality using MELD score represented as area under the curve (AUC) was 0.82 (95 % confidence intervals (CI): 0.65–0.98), and AUC of DF was 0.86 (95 % CI: 0.70–1.00). Sheth et al. therefore suggested that treatment for AH should be considered when MELD score is greater than 11.

Other studies have suggested higher cut-offs for MELD scores for predicting mortality. In a retrospective study, Srikureja et al [22] reported that MELD score is better than DF in predicting in-hospital mortality. An admission MELD score ≥18 showed a sensitivity and specificity of 85 % and 84 %, respectively, for predicting in-hospital mortality. First week MELD score ≥20 had a 91 % sensitivity and 85 % specificity, and first week change of MELD score ≥2 points had a 80 % sensitivity and 75 % specificity for predicting in-hospital mortality. Dunn et al. [17], in a retrospective review of 73 patients showed that MELD score was the only independent predictor of mortality in patients with AH. MELD was shown to be comparable to DF in predicting 30-day and 90-day mortality. A MELD score of 21 in the study was shown to have a sensitivity of 75 % and a specificity of 75 % to predict mortality with an estimated 90-day mortality of 20 % in AH. Another study reported a 30-day and 90-day mortality rates of 5.9 % and 14.7 %, respectively in patients with AH. MELD score >30.5 had an excellent performance in predicting 30-day mortality with a sensitivity of 100 % and specificity 94 % (AUC 0.969). Furthermore, MELD score >19 only had a fair performance in predicting 90-day mortality with a sensitivity of 60 % and a specificity of 60 % (AUC 0.762) [23].

In a retrospective study of 26 patients, Vaa et al [24] compared MELD and MELD-Na in predicting 180-day mortality in patients with AH. MELD-Na is a modified MELD score that includes serum sodium (Na) which has been shown to improve prediction of death in cirrhotic patients. In the study, MELD-Na was a better predictor of 180-day mortality than MELD in patients with ascites. A MELD score of 27 and a MELD-Na score of 28 in patients without ascites had a sensitivity/specificity of 76.5 %/64.9 % and 87.5 %/52.5 %, respectively. After adjustment for MELD in AH patients without ascites, serum Na, specifically hyponatremia, was not a significant predictor of mortality (*p* = 0.83). However, in patients with ascites a MELD score of 29 and a MELD-Na score of 34 had sensitivity/specificity of 85.7 %/31.0 % and 83.3 %/16.7 %, respectively. In AH patients with ascites, MELD-Na had better predictability of 180-day mortality compared to MELD (odds ratio (OR), 2.27 for every 1-point increase in MELD-Na; 95 % CI: 1.22–36.68; *p* = 0.008 versus OR, 1.37 for every 1-point increase in MELD; 95 % CI: 1.07–2.12; *p* = 0.006).

Overall MELD score is a good predictor for mortality in patients with AH. According to therapeutic algorithms, an initial MELD score ≥18 and increasing serial MELD scores over time are considered high risk for mortality and should be used in guiding initiation of therapeutic intervention [2].

### Glasgow alcoholic hepatitis score (GAHS)

In 2005, Forrest et al. [25] used 5 variables including age, blood urea, peripheral blood leukocyte count, serum bilirubin, and PT, expressed as a ratio of the control value to develop a new prognostic scoring system for AH. Values obtained ranged from 5 to 12, separated into those with value <9 or ≥9 points. Day 28 survival in patients with a day 1 GAHS score of <9 was 87 % compared to 46 % in those with GAHS ≥9. Based on the validation dataset, accuracy of GAHS day 1 and 7 data had a better prediction of 28 day and 84 day outcome than mDF. Accuracy of GAHS day 1 and 7 in predicting 28-day mortality was 67 % and 75 %, versus mDF accuracy of 48 % and 56 %; *p* = 0.0016 and *p* = 0.0038, respectively. Accuracy of GAHS day 1 and 7 in predicting 84-day mortality was 71 % and 75 %, versus mDF accuracy of 57 % and 62 %; *p* = 0.0179 and *p* = 0.0477, respectively. In a subsequent study in 2007 by Forrest et al. [26], patients with a mDF ≥32 and a GAHS <9 did not benefit from treatment with corticosteroids. However corticosteroids therapy was associated with better survival in those with both GAHS ≥9 and mDF ≥32 compared to no treatment (28-day survival 78 % vs. 52 %, *p* = 0.002). Day 84 survival was 59 % and 38 % (*p* = 0.02) in those treated and not treated with steroids, respectively. In a recent study, Lafferty et al. [27] showed that patients receiving corticosteroids with a GAHS ≥9, irrespective of mDF, had a 90-day survival of 58 % compared to 30 % in those not receiving treatment. The sensitivity and specificity of 90-day outcome for GAHS assessment on admission was 67 % and 78 %, respectively.

### Age, serum bilirubin, INR, and serum creatinine (ABIC) score

In 2008, Dominguez et al. [28] built a predictive score from multivariate analysis of variables identified during admission. The resulting score: Age, serum Bilirubin, INR, and serum Creatinine (ABIC) score = (age × 0.1) + (serum bilirubin × 0.08) + (serum creatinine × 0.3) + (INR × 0.8) was validated in an independent prospective cohort. Using a cutoff value of 6.71 and 9 the score identified patients with AH that have a low (100 % survival), intermediate (70 % survival), and high risk (25 % survival) of death at 90 days. The sensitivity and specificity was 100 % and 50 % for the cut-off of 6.71, respectively, and 70 % and 33 %, respectively, for a cut-off of 9. In a retrospective study evaluating 9 different scoring systems, the sensitivity and specificity of the ABIC score for 30-day mortality were 100 % and 20 %, respectively, for a cut-off of 6.71, and 60 % and 80 %, respectively, for a cut-off of 9. A higher ABIC cut-off of 9.5 compared to original cut-off of 9 resulted in an increased specificity of 90 % vs. 80 % and 95 % vs. 84 % for a 30- and 90-day outcome respectively [16]. When comparing the 90-day mortality predictive accuracy of the ABIC score with MELD, mDF, and GAHS, the ABIC score was the best independent predictor of 90-day mortality (hazard ratio (HR) 2.78, 95 % CI 1.90–4.09, *p* = 0.0001). The ABIC score was also assessed to determine 1-year mortality, which could be used for liver transplantation assignment. The ABIC score was the only independent predictor of 1-year mortality (HR: 2.49, 95 % CI 1.77–3.52, *p* = 0.0001), when compared to other prognostic models. Analysis of the subgroup of patients treated with steroids showed that the greatest response was in the group with intermediate ABIC score (between 6.71–8.99) compared to those with either low ABIC (<6.71) or high ABIC scores (>9). In patients treated with steroids, the ABIC score at 7 days has a better accuracy than the Lille model in predicting mortality at 6 months [28].

### Lille model

In 2007, Louvet et al. [29] generated a prognostic model, the Lille model, to identify “non-responders” to corticosteroid therapy in patients with severe AH. The model combined six objective variables (age, renal insufficiency, albumin, PT, bilirubin, and evolution of bilirubin at day 7) which were highly predictive of death at 6 months in patients treated with corticosteroids (p < 0.000001). The Lille formula is available online at http://www.lillemodel.com or see formula in Table 1. A cut-off value of 0.45 was determined to be the best identifier of patients at high risk of death, with a sensitivity and specificity of 81 % and 76 %, respectively in the validation cohort, and 76 % and 85 %, respectively on overall patients. Patients with a Lille score ≥0.45 had a significant decrease in 6-month survival compared to patients with a Lille score <0.45 (25 % versus 85 %; p *<* 0.0001). Thus, 40 % of patients receiving steroids can be identified to have a poor prognosis using the Lille model. Patients receiving corticosteroids after 7 days with a score ≥0.45 may be futile and alternative treatments should be considered. In a prospective study regarding infection in AH patients, only the Lille model (OR, 11.14; 95 % CI: 3.2–39.2, *p* = 0.0002) independently predicted infection upon steroids use in multivariate analysis. Responders (Lille model <0.45) to steroids that developed infection had lower survival compared to responders that did not develop infection: 51 % vs 94 %, respectively (*p* = 0.000001). Non-responders (Lille model ≥0.45) that developed infection had similar survival to non-responders that did not develop infection: 42 % vs 52 %, respectively (*p* = 0.5). Thus adjuvant antibiotic therapy to corticosteroids in the setting of severe AH may improve survival mainly in responders [30].

**Table 1:** Prognostic scoring formulas to determine severity of acute alcoholic hepatitis

Scoring system	Formula			Severe Disease	
mDF	4.6 (patient’s PT in seconds- control PT in seconds) + total bilirubin (mg/dL)			≥32	
CTP	1	2	3		
Bilirubin	<2 mg/dL	2–3 mg/dL	>3 mg/dL	Class A	5–6 points
Albumin	>3.5 g/dL	2.8–3.5 g/dL	<2.8 g/dL	Class B	7–9 points
INR	<1.7	1.7–2.2	>2.2	Class C	10–15 points
Ascites	None	Mild	Severe		
Encephalopathy	None	Grade I-II	Grade III-IV		
MELD	MELD Score = 0.957 x Loge (creatinine mg/dL) + 0.378 x Loge (bilirubin mg/dL) + 1.120 x Loge (INR) 0.6431* 10 (if hemodialysis, value for Creatinine is automatically set to 4.0)			MELD ≥ 21	
	MELD score = 3.8*loge (total bilirubin, mg/dL) + 11.2*loge (INR) + 9.6*loge(creatinine, mg/dL)				
	MELD score = 9.57 x loge (Cr mg/dL) + 3.78 x loge (bili mg/dL) + 11.20 x loge (INR) + 6.43				
MELD-Na	MELD-Na score = MELD Score - Na - 0.025*MELD* (140-Na) + 140				
GAHS	1	2	3	≥9	
Age	<50	≥50	-		
WBC (109/l)	<15	≥15	-		
Urea (mmol/l)	<5	≥5	-		
PT ratio	<1.5	1.5–2.0	>2.0		
Bilirubin (μmol/l)	<125	125 - 250	>250		
Lille Score	Lille Score = 3.19–0.101 * (age in years) + 0.147 * (albumin day 0 in g/L) + 0.0165 * (evolution in bilirubin level in μM) - (0.206 * renal insufficiency) - 0.0065 * (bilirubin day 0 in μM) - 0.0096 * (INR or prothrombin time in seconds).			≥0.45	
ABIC score	ABIC score = (age × 0.1) + (serum bilirubin × 0.08) + (serum creatinine × 0.3) + (INR × 0.8)			>9.0	
AHHS			Points		
	Stage of fibrosis			Mild (0–3)	
	No fibrosis or portal fibrosis		0	Moderate (4–5)	
	Expansive fibrosis		0	Severe (6–9)	
	Bridging fibrosis or cirrhosis		3		
	Bilirubinostasis				
	No		0		
	Hepatocellular only		0		
	Canalicular or ductular		1		
	Canalicular or ductular plus hepatocellular		2		
	PMN infiltration				
	No/ Mild		2		
	Severe		0		
	Megamitochondria				
	No Megamitochondria		2		
	Megamitochondria		0		

*mDF* modified Discriminant Function, *CTP* Child Turcotte Pugh, *MELD* Model for End-stage Liver Disease, *GAHS* Glasgow Alcoholic Hepatitis Score, *ABIC* Age serum Bilirubin INR and serum Creatinine, *AHHS* Alcoholic Hepatitis Histologic Score

### Beclere model

The Beclere model was initially formulated by Poynard et al. [31] in 1994 to determine survival in alcoholic cirrhosis. The final model has four variables: age, encephalopathy, serum bilirubin, and serum albumin to obtain a risk score (R) for each patient using the formula R = (0.0484 × [Age in Years] + 0.469 × [encephalopathy] + 0.537 × Log_e_ [Bilirubin in μmol/L] - 0.052 × [Albumin in g/L]. This model was then used by Mathurin et al. [32] as a simulated prognostic model for a control group in a study aimed to examine prognostic factors and long-term survival in AH patients receiving corticosteroids. There was no difference in 1 and 2 year survival in the observed placebo-randomized group and the simulated control group using the Beclere model. In a multivariate analysis, the corticosteroid effect (p < 0.02) and the Beclere model risk score (*p* = 0.0003) had independent prognostic value for survival. Survival was significantly better in the treated groups compared to the non-treated groups. The prednisolone - randomized group had 69 % survival (95 % CI: 57 %–81 %) and 71 % (95 % CI: 55 %–87 %) in the prednisolone-open group, compared to 41 % (95 % CI: 23 %–59 %; *p* = 0.01) in the placebo-randomized group and 50 % (95 % CI: 37 %–63 %; *p* = 0.05) in the simulated control group. However, subsequent studies in AH patients did not further use this model for prognostic scoring.

### Alcoholic hepatitis histologic score (AHHS)

The need for liver biopsy is controversial in diagnosing alcoholic hepatitis patients as the presence of ascites and coagulopathy may require a transjugular approach, which may not be readily available [33]. Altamirano et al. [34] developed a histologic scoring system based on liver biopsy findings to predict short-term (90-day) mortality in AH patients. The AHHS was initially developed from 121 patients admitted to a single center in Spain, and subsequently tested and validated in another set of 205 patients from 5 academic centers in the United States and Europe. After multivariate analysis, four independent histologic features were combined in the final score: fibrosis stage (0–3) which was separated as bridging fibrosis or cirrhosis giving a score of +3 versus a score of 0 for absence of these features; bilirubinostasis (0–2) was divided into a score of 0 for absence or hepatocellular only, +1 for canalicular or ductular, and +2 for canalicular or ductular plus hepatocellular; polymorphonuclear (PMN) infiltration (0–2) was described as “mild” PMN (score +2) when usually <15 PMN per focus were found around a hepatocyte and “marked” PMNs were easily recognized at low magnification and many PMNs found around damaged hepatocytes (with ballooning or Mallory–Denk bodies); and megamitochondria (0–2) where none seen was +2, and seen was 0, for a total of 9 points. Marked PMN infiltration and megamitochondria were independently associated with a favorable outcome. AHHS cut-off score categorized patients as low 0–3 (97 % survival), intermediate 4–5 (81 % survival), and high risk 6–9 (49 % survival) of death. When combing the AHHS with analytical scoring systems, the AHHS was able to refine the prognostic stratification of those with a MELD score <21 (low risk group). In patients with a MELD score <21 (low risk group) the AHHS was able to define 2 subgroups with different 90-day survival using a cut-off of 5 points (94 % vs 72 %; *p* = 0.001). The differences in the MELD with an AHHS <5 points and those with an AHHS ≥5 points (16 ± 8 vs 23 ± 9 points of MELD, respectively; p < 0.0001) suggest that analytic parameters in the MELD score, such as bilirubin, are reflected in severity of histologic abnormalities. This would, therefore, modify stratification of severity from a low to high risk group, and change treatment management in these patients.

### Child–Turcotte–Pugh (CTP) score

The CTP is based on 5 variables including ascites, encephalopathy, serum bilirubin, albumin and PT, which the latter was modified in 1973 by Pugh et al. from the original use of nutritional status in the Child-Turcotte criteria. Each variable has a score of 1 to 3, and patients are classified as class A (best), B (moderate), or C (worse) to determine prognosis, originally for cirrhotic patients undergoing surgery [35,36]. The limitations to the scoring system include subjectivity in ascites and encephalopathy grading, variable PT results, and a “ceiling” and “floor” effect for arbitrary cut-off points with bilirubin and albumin, respectively [37]. The use of CTP score in AH is not widely used. In 2004, Said et al [38] reported that CTP compared to the MELD score had similar predictive abilities for 3- and 6-month mortality in AH patients, with a c-statistics of 0.85 (0.75–0.95) and 0.81 (0.70–0.92), respectively. Srikureja et al. [22] retrospectively compared MELD, CTP, and DF scores as predictive models to assess in-hospital mortality in AH patients. CTP score was independently associated with mortality on admission with an AUC of 0.87 (95 % CI: 0.81–0.94). However, this was lost with the first week change in CTP score (AUC 0.57; 95 % CI: 0.43–0.70) compared to first week change in MELD score (AUC 0.85; 95 % CI: 0.76–0.94; *p* = 0.0004).

### TMA and pentane (TAP) score

A recent study by Hanouneh et al. [39] identified novel breath biomarkers in patients with AH. Six compounds including 2-propanol, acetaldehyde, acetone, ethanol, pentane, and trimethylamine (TMA) were initially shown to be increased in patients with liver disease compared with healthy control subjects. TMA, acetone and pentane were significantly higher in AH compared to those with acute decompensation or control subjects (for all, p < 0.001). After accounting for MELD score only the associations between TMA and pentane (TAP) to AH remained significant (TMA, p < 0.001; pentane, *p* = 0.004). Combining pentane and TMA levels in the breath was found to have an excellent prediction accuracy to diagnose AH (AUC 0.92). A model, named the TAP score, was then developed using the logistic regression (lr) function of the two variables (lr = −3.71 + [0.34*TMA] - [0.087*pentane]), and the following derived formula, TAP score = 100 × (exp [lr]/1 + exp [lr] was used. TAP scores of ≥36 identified patients with AH with 90 % sensitivity and 80 % specificity. There correlation of the so called breathprint and severity of liver disease was only moderate as presented by MELD score (r = 0.38; 95 % CI, 0.07–0.69; *p* = 0.18). Larger studies are needed to further validate these results, as only a small group of 40 patients with AH were assessed.

### Combining static and dynamic models

Louvet et al. [40] evaluated the prognostic value of combining static models for AH, such as mDF, MELD score, and ABIC score with dynamic models, such as the Lille score. This joint effect model was able to predict outcome of survival after 2 and 6 months significantly better than either the static or dynamic models alone (p < 0.01). The MELD + Lille combination model predicted survival better than the mDF + Lille or ABIC + Lille models. Using the joint effect model of MELD + Lille score, a hypothetical patient with a MELD score of 21 and Lille score at 0.45 had a 15.3 % and 23.7 % mortality rate at 2 and 6 months, respectively. The overall predicted mortality at 6 months in the MELD + Lille model with a MELD score of 15–45 was between 8.5 %–49.7 % in a complete responder (Lille score, 0.16), and from 16.4 %–75.2 % in a non-responder (Lille score, 0.45). The use of the joint effect models has a better prediction of mortality in AH patients. This models can also identify high risk of death in patients previously classified as responders, and intermediate risk of death in previous classified non-responders.

### Further indicators of prognosis in alcoholic hepatitis

#### Acute kidney Injury (AKI)

AKI, as per the AKIN (Acute Kidney Injury Network) criteria, is defined as an abrupt reduction (within 48 h) in kidney function that results in an absolute increase of at least 0.3 mg/dL (or a 50 % increase) in serum levels of creatinine from baseline [41]. In a retrospective study by Altamirano et al [42], AKI was shown to markedly influence 90 day mortality in patients with AH versus without AH (65 % vs. 7 %, p < 0.0001). The most accurate predictors of AKI were the presence of systemic inflammatory response syndrome, serum bilirubin (especially >16 mg/dL), and elevated INR >1.7 in patients with AH.

#### Change in bilirubin

Similar to the Lille score, early change in bilirubin levels (ECBL), defined as bilirubin level at 7 days lower than bilirubin level on the first day of treatment of steroids was shown to be an important prognostic factor in AH patients. Mathurin et al [43] reported that 95 % of patients with ECBL continued to have improved liver function during treatment. Six-month survival in patients with ECBL was 82.8 ± 3.3 % versus 23 ± 5.8 % (p < 0.0001) in the non-ECBL group. Another study by Morris and Forrest [44] identified steroid responders, defined as a 25 % fall in serum bilirubin after 6–9 days of treatment had a better survival than non-responders. Non-responders were found to have 28-day and 56-day mortality of 36.8 % and 57.9 %, respectively, versus responders with 0 % (*p* = 0.0148) and 11.1 % (*p* = 0.0084), respectively. The above studies, therefore, suggest stopping steroids in non-responders.

#### Gastrointestinal (GI) bleed

In a recent study, Rudler et al. [45] compared the mortality risk in AH patients who had concomitant GI bleeding (AH-GIB+) with AH patients without GI bleeding (AH-GIB-). There was no difference in 1, 3, and 6-month probability of survival in AH-GIB+ versus AH-GIB- groups (87.9 ± 4 % vs 84.1 ± 5 %, *p* = 0.56; 79.2 ± 5 % vs 71.1 ± 7 %, *p* = 0.24; and 73.9 ± 6 % vs 69.9 ± 7 %, *p* = 0.49, respectively). There was also no difference in response to therapy between the two groups as well.

#### Protein-Calorie malnutrition

Malnutrition, to some degree is found in every AH patient whether they do or do not have concomitant cirrhosis [46]. Mendenhall et al. [47] found that the severity of protein energy malnutrition (PEM) correlated with prognosis of AH patients. A moderate PEM of 60 %–79 % of normal was associated with a 29 % 6-month mortality, and severe malnutrition (PEM score <60 %) correlated with a 45 % mortality in 6 months. Patients with moderate malnutrition and adequate caloric intake (>2,500 kcal/day) that were given oxandrolone (an anabolic steroid) had a reduced 6-month mortality of 4 % versus 28 % in the placebo group (*p* = 0.002). These findings were not seen in cases of severe malnutrition [48]. In a randomized control trial, AH patients received either prednisone or total enteral nutrition (TEN) of 2,000 kcal/day for 28 days [49]. Short term (28-day period) mortality was 25 % and 31 % in the steroid group and TEN group, respectively. One-year survival probability was 39 % with steroids and 62 % with TEN. Thus there may be a synergistic effect in using steroid and TEN in AH treatment. In a meta-analysis, Antar et al. [50] pooled 7 randomized control trials that compared nutritional supplementation plus a normal hospital diet versus diet alone. There was no statistical difference in mortality between the two groups (OR, 0.80; 95 % CI: 0.42–1.52), although there was a trend toward survival benefit with supplemental nutrition and significant improvement in encephalopathy (OR, 0.24; 95 % CI: 0.06–0.93). Thus nutritional supplement is still regarded as beneficial.

## Conclusion

The available scoring systems are useful in both prognostic stratification and selection of candidates for appropriate therapy. Limitations of the scoring systems include differing cut-offs to meet the best accuracy, clinical and laboratory parameters that may differ, and that one scoring system may be insufficient to determine some patients with severe AH. Combining more than one scoring system, e.g. DF and Lille score, MELD and AHHS, or static and dynamic models may better define severe AH with greater accuracy. Furthermore, determining responders versus non-responders to therapy is important to avoid untoward adverse events associated with treatment.

## Abbreviations

AH: Alcoholic hepatitis
AST: Serum aspartate aminotransferase
ALT: Alanine aminotransferase
AASLD: American Association for the Study of Liver Diseases
DF: Discriminant function
PT: Prothrombin time
mDF: Modified DF
MELD: Model for End-Stage Liver Disease
INR: International normalized ratio
TIPS: Transjugular intrahepatic portosystemic shunts
AUC: Area under the curve
CI: Confidence intervals
OR: Odds ratio
GAHS: Glasgow Alcoholic Hepatitis Score
ABIC: Age serum Bilirubin INR and serum Creatinine
HR: Hazard ratio
AHHS: Alcoholic Hepatitis Histologic Score
PMN: Polymorphonuclear
CTP: Child–Turcotte–Pugh
AKI: Acute Kidney Injury
AKIN: Acute Kidney Injury Network
ECBL: Early change in bilirubin levels
GI: Gastrointestinal
PEM: Protein energy malnutrition
TEN: Total enteral nutrition
